# A randomized phase II study of SRL172 (*Mycobacterium vaccae*) combined with chemotherapy in patients with advanced inoperable non-small-cell lung cancer and mesothelioma

**DOI:** 10.1054/bjoc.2000.1401

**Published:** 2000-09-04

**Authors:** M E R O’Brien, A Saini, I E Smith, A Webb, K Gregory, R Mendes, C Ryan, K Priest, K V Bromelow, R D Palmer, N Tuckwell, D A Kennard, B E Souberbielle

**Affiliations:** Royal Marsden Hospital NHS Trust, Sutton, Surrey SM2 5PT; Kent Cancer Centre, Maidstone, ME16 9QQ; Dept of Molecular Medicine, King’s College, London, SE5 9NU; Department of Bacteriology, University College London Medical School, W1P 6DB; SR Pharma, 26th Floor, Centre Point, 103 New Oxford Street, London, WC1A 1DD, UK

**Keywords:** lung cancer, mesothelioma, SRL172, mycobacterium vaccae, chemotherapy

## Abstract

Mycobacterial preparations have been used with limited success against cancer apart from superficial bladder cancer. Recently, a therapeutic vaccine derived from *Mycobacterium vaccae* has been given to patients with prostate cancer and melanoma indicating a possible beneficial effect on disease activity in such patients. We have recently initiated a series of randomized studies to test the feasibility and toxicity of combining a preparation of heat-killed *Mycobacterium vaccae* (designated SRL172) with a multidrug chemotherapy regimen to treat patients with inoperable non-small cell lung cancer (NSCLC) and mesothelioma. 28 evaluable patients with previously untreated symptomatic NSCLC and mesothelioma were randomized to receive either 3 weekly intravenous combination chemotherapy alone, or chemotherapy given with monthly intra-dermal injections of SRL172. Safety and tolerability were scored by common toxicity criteria and efficacy was evaluated by survival of patients and by tumour response assessed by CT scanning. The toxicity of chemotherapy was similar in the two groups. SRL172 caused mild inflammation at the injection site. In the group of patients randomized to receive chemotherapy combined with SRL172, there was a trend towards improved response rate (54% vs. 33%) with more patients in the combined arm receiving radical surgery and radiotherapy, improved median survival (9.7 months vs. 7.5 months) and improved 1 year survival (42% vs. 18%). SRL172 appeared to improve sleep (*P* = 0.08) and improved appetite (*P* = 0.01). There was no detectable change in serum cytokine levels for gamma-interferon and TNF-α before and after treatment. In patients with NSCLC and mesothelioma, there may be a beneficial interaction when chemotherapy is administered in combination with SRL172. Confirmation of this effect and further investigation is underway in a randomized phase III trial and in laboratory models. © 2000 Cancer Research Campaign


British Journal of Cancer (2000) 83(7), 853–857
© 2000 Cancer Research Campaign
doi: 10.1054/ bjoc.2000.1401, available online at http://www.idealibrary.com on IDEAL®

### A randomized phase II study of SRL172 (*Mycobacterium vaccae*) combined with chemotherapy in patients with advanced inoperable non-small-cell lung cancer and mesothelioma

**MER O'Brien[1,2], A Saini[1], IE Smith[1], A Webb[1], K Gregory[1], R Mendes[1,3], C Ryan[2], K Priest[1], KV Bromelow[3], RD Palmer[4], N Tuckwell[6], DA Kennard[5] and BE Souberbielle[1,3]**

[1]Royal Marsden Hospital NHS Trust, Sutton, Surrey, SM2 5PT; [2]Kent Cancer Centre, Maidstone, ME16 9QQ; [3]Dept of Molecular Medicine, King's College, SE5 9NU, London, [4]Department of Bacteriology, University College London Medical School, W1P 6DB; [5]SR Pharma, 26th Floor, Centre Point, 103 New Oxford Street, London WC1A 1DD, UK

**Summary** Mycobacterial preparations have been used with limited success against cancer apart from superficial bladder cancer. Recently, a therapeutic vaccine derived from *Mycobacterium vaccae* has been given to patients with prostate cancer and melanoma indicating a possible beneficial effect on disease activity in such patients. We have recently initiated a series of randomized studies to test the feasibility and toxicity of combining a preparation of heat-killed *Mycobacterium vaccae* (designated SRL172) with a multidrug chemotherapy regimen to treat patients with inoperable non-small cell lung cancer (NSCLC) and mesothelioma. 28 evaluable patients with previously untreated symptomatic NSCLC and mesothelioma were randomized to receive either 3 weekly intravenous combination chemotherapy alone, or chemotherapy given with monthly intra-dermal injections of SRL172. Safety and tolerability were scored by common toxicity criteria and efficacy was evaluated by survival of patients and by tumour response assessed by CT scanning. The toxicity of chemotherapy was similar in the two groups. SRL172 caused mild inflammation at the injection site. In the group of patients randomized to receive chemotherapy combined with SRL172, there was a trend towards improved response rate (54% vs. 33%) with more patients in the combined arm receiving radical surgery and radiotherapy, improved median survival (9.7 months vs. 7.5 months) and improved 1 year survival (42% vs. 18%). SRL172 appeared to improve sleep ($P = 0.08$) and improved appetite ($P = 0.01$). There was no detectable change in serum cytokine levels for gamma-interferon and TNF-$\alpha$ before and after treatment. In patients with NSCLC and mesothelioma, there may be a beneficial interaction when chemotherapy is administered in combination with SRL172. Confirmation of this effect and further investigation is underway in a randomized phase III trial and in laboratory models. © 2000 Cancer Research Campaign

**Keywords:** lung cancer; mesothelioma; SRL172; mycobacterium vaccae; chemotherapy

500 000 new cases of lung cancer are diagnosed world-wide annually. 80% of these are non-small-cell type (NSCLC). Pleural mesothelioma is a less common cancer arising from the serosa of the lung, the incidence of which is closely linked to asbestos exposure. The response rates to conventional chemotherapy for both these cancers ranges from 20% to 50%. Chemotherapy gives symptom palliation and improvement in quality of life for patients with these tumours and a small but real and reproducible improvement in survival for NSCLC tumours (Ellis et al, 1995). Over the last 10 years, many new drugs with novel mechanisms of action have shown activity against lung cancer, but the survival rates are comparable to established agents (Webb and O'Brien, 1998). There remains therefore a need for new strategies, one of which is the re-exploration of immunotherapy. Therapeutic vaccination using non-specific immunostimulants, e.g. intradermal BCG, has been tested in randomized clinical trials in lung cancer, but a clear benefit has never been shown for its use (Al-Moundhri et al, 1998).

*Received 2 December 1999*
*Revised 29 May 2000*
*Accepted 22 June 2000*

*Correspondence to:* MER O'Brien

The hypothesis leading to this study was that chemotherapeutic agents could induce in vivo release of either tumour-specific or tumour-associated antigens from the treated tumour and that a non-specific immunostimulator could concomitantly boost tumour antigen recognition in patients who may be immuno-suppressed from their cancer. SRL172 is a suspension of heat-killed *Mycobacterium vaccae* which is currently undergoing clinical evaluation in humans. Immunological effects have been observed in patients with prostate cancer and melanoma (Hrouda et al, 1998; Maraveyas et al, 1999). Randomized phase II trials to study the clinical and immunological effects of SRL172 in patients with cancer have been carried out at the Royal Marsden Hospital, with a view to developing it as an immunological adjuvant for tumour treatments. In this study, the feasibility, toxicity and clinical effects of combining intradermal SRL172 with intravenous chemotherapy were assessed in a cohort of patients presenting with advanced, inoperable lung cancer or mesothelioma.

### PATIENTS AND METHODS

29 previously untreated patients with histologically or cytologically verified symptomatic NSCLC, or mesothelioma, were randomized to receive either intradermal SRL172 given concurrently with intravenous combination chemotherapy, or chemotherapy alone. This was the NSCLC part of a larger phase II study using the same design for SCLC comparing chemotherapy to chemotherapy with SRL 172 (28 patients), and in asymptomatic patients with NSCLC and mesothelioma comparing best supportive care (BSC) to BSC with injections of SRL 172 (28 patients). The latter 2 components of this trial will be reported within the next 12–18 months. All patients had lesions evaluable by clinical or radiological examination. Other inclusion criteria were life-expectancy of 12 weeks or more, age over 18 years and satisfactory renal function. Patients whose performance status was 3 or more, or who had serious concomitant illness were excluded. The protocol was approved by the Research Ethics Committees of The Royal Marsden NHS Trust and the Kent Cancer Centre. Written informed consent was obtained from all patients. All patients underwent pre-treatment physical examination, plasma electrolytes, urea and creatinine, serum liver function tests, chest radiography and staging of disease with a thoraco-abdominal CT scan. Clinical assessment of symptoms took place at the time of randomization, monthly while receiving injections of SRL172 and thereafter at 3–6 monthly intervals. Quality of life data collection was planned at baseline and at 3 monthly intervals, patients were in addition asked specifically in one question if their sleep pattern had changed and if their appetite had changed. At each assessment visit, patients had physical examination, FBC and chest radiography. Objective response was measured by CT scanning every two courses.

15 patients received standard combination chemotherapy alone (mitomycin C 8 mg/m$^2$ on course 1, 2, 4 and 6, vinblastine 6 mg/m$^2$ (maximum dose 10 mg) and cisplatin 50 mg/m$^2$) and 14 patients received the same chemotherapy combined with SRL172. Chemotherapy was given at 3 weekly intervals for a maximum of 6 courses and SRL172 was given intradermally prior to the first chemotherapy and then at monthly intervals for the first 3 injections, then at 3–6 monthly intervals thereafter. Antiemetics were routinely prescribed but dexamethasone was omitted for the first course in those who received SRL172.

SRL172 was formulated as a suspension of 10 mg/ml heat killed *Mycobacterium vaccae* in borate buffered saline (pH 8) and provided in 3 ml glass vials at a concentration of $10^9$ bacilli per 0.1 ml dose and stored refrigerated at 4°C in the pharmacies of both hospitals taking part in the trial.

Serum cytokine levels of TNF-alpha, gamma-interferon and IL-10 were measured by ELISA pre- and post-treatment (1 month after the end of the chemotherapy) using the supplier protocol (R&D Systems, Abingdon, UK).

### RESULTS

Patient characteristics are shown in Table 1. The two groups were well balanced with regard to performance status, age and sex, but there were slightly more patients with adenocarcinoma in the chemotherapy alone group (2 versus 5). One patient in the combined treatment arm was not eligible for entry into the study because of the presence of renal failure and ongoing immunosuppressive therapy prior to treatment.

There were 20 male and 9 female patients. WHO performance status (PS) was as follows; 9 patients were PS 1, 19 patients were PS 2, 1 patient was PS 3. A total of 9 patients had mesothelioma and 20 had NSCLC. The majority of patients were stage IIIB or stage IV, with 2 patients stage IIIA. The median age was 60 years (range 33–71 years).

At the time of randomization, all patients had symptoms requiring treatment. These consisted of cough, dyspnoea and/or pain. Following injection of SRL172, there was no treatment related toxicity, except for some induration at the injection site. In the group treated with chemotherapy plus SRL172, on the intention to treat analysis (ITT) there was a trend towards an improved median survival was 9.4 months vs 7.5 months ($P$ = 0.3), and looking at the patients evaluable for response ($n$ = 28) there was also a trend towards improved response rate (54% vs. 33%, $P$ = 0.3), improved median survival (9.7 months vs. 7.5 months, $P$ = 0.235) and in the proportion of treated patients alive at one year (42% vs. 18%) compared with the group receiving chemotherapy alone (Figure 1: intention to treat survival analysis). Two patients in the combined treatment arm subsequently underwent potentially curative surgery and one patient had radical radiotherapy.

Treatment toxicity is shown in Tables 2 and 3 and was comparable in the two groups of patients. SRL172 appeared to improve

**Table 1** Patient characteristics

| | | Chemotherapy + SRL172 | Chemotherapy |
|---|---|---|---|
| Sex | F/M | 3/11 | 6/9 |
| Age | | 61 | 59 |
| Range | | 33–71 | 40–70 |
| PS | 1 | 3 | 6 |
| | 2 | 10 | 9 |
| | 3 | 1 | 0 |
| Stage | IIIa | 1 | 1 |
| | IIIb | 7 | 6 |
| | IV | 6 | 8 |
| Path | adeno | 2 | 5 |
| | squamous | 2 | 2 |
| | adenosquamous | 1 | 0 |
| | large cell | 0 | 1 |
| | unclassified | 5 | 2 |
| | mesothelioma | 4 | 5 |
| No previous radiotherapy | | 12 | 14 |
| Weight loss in past 3 mths | | | |
| | < 5% | 11 | 10 |
| | 5+% | 3 | 5 |

*© 2000 Cancer Research Campaign*

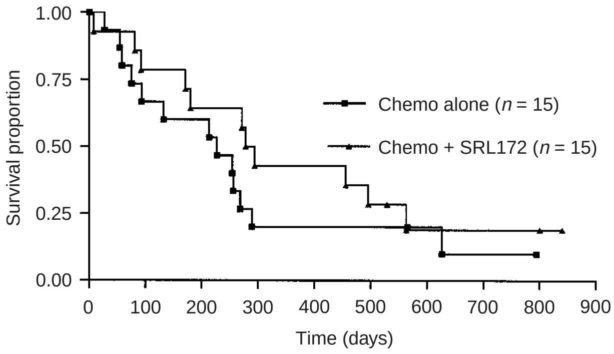

**Figure 1.** Survival analysis (intention to treat)

**Table 2** Haematological toxicity (% of cycles at any time during study period)

| | Chemotherapy + SRL172 *n* = 50 | Chemotherapy *n* = 57 |
|---|---|---|
| Haemaglobin | | |
| Abnormal | 74% | 65% |
| G3,4 | 0% | 0% |
| Neutrophils | | |
| Abnormal | 6% | 21% |
| G3,4 | 2% | 2% |
| Lymphocytes | | |
| Abnormal | 52% | 56% |
| Platelets | | |
| Abnormal | 14% | 33% |
| G3,4 | 0 | 0 |

**Table 3** Non-haematological toxicity (Grade 3, 4 total number on any cycle)

| | Chemotherapy +SRL172 | Chemotherapy |
|---|---|---|
| Infection | 3* | 0 |
| Nausea and vomiting | 5 | 1 |
| Mucositis | 0 | 0 |
| Diarrhoea | 0 | 0 |
| Constipation | 0 | 0 |
| Alopecia | 2 | 0 |
| Nephrotoxicity | 1 | 1 |
| Malaise | 2 | 3 |
| Poor appetite | 2 | 13 (*P* = 0.01) |
| Insomnia | 5 | 14 (*P* = 0.08) |

* One toxic death sleep (*P* = 0.08) and improved poor appetite (*P* = 0.01). Full quality of life assessment has not been analysed as the numbers of patients returning repeat questionnaires was small. There was one treatment related death in the group receiving SRL172 and chemotherapy. A patient with Poland's syndrome (congenital absence of the chest wall) with a stage IV adenocarcinoma developed a chest infection after 4 courses of chemotherapy. Initially he was neutropenic but this quickly resolved. His disease was responding on assessment of the measurable mass on his sternum. The lung cancer was on the R side – the side of this absent chest wall, the infection was mostly on this side also. We felt the cause of death was a chest infection in this hypoventilated lung but underlying progressive disease could have contributed. Permission to do a post-mortem was not given after his death. Of the patients with mesothelioma, 2/4 had PR in the combination group compared with 1/5 in the chemotherapy alone group.

Serum cytokine levels of TNF-α, γ-interferon (γ-IFN) and IL-10 were measured by ELISA (R&D systems) pre- and post-treatment at 1 month after the end of the chemotherapy (Table 4). There was no statistical difference between the 2 treatment groups (chemotherapy and chemotherapy + SRL 172) at baseline for the γ-IFN and TFN-α serum levels (*P* = 0.2 and *P* = 0.9 respectively, two-tailed *P* value, Mann-Whitney U-test). Baseline levels were also compared between responders and non-responders irrespective of the treatment group as possible prognostic factors. There was no difference in the serum levels of γ-IFN (*P* = 0.76, Mann-Whitney U test, 2 tailed *P* value) but the serum levels of TNF-α at baseline were lower in the responders group (mean 37.9 pg/ml) compared to the non-responders group (mean 45.8 pg/ml), (*P* = 0.01, two-tailed *P* value, Mann-Whitney U-test). However the *P* value became non-significant when it was corrected for the number of comparisons done in the study (× 14) using the Bonferoni correction: *P* = 0.14. In addition there was no difference, when baseline cytokine levels were compared to post-treatment levels (*P* values are shown in the last column of Table 3). IL-10 was detected in the serum of only 30% of patients (the IL-10 serum levels of the rest of the patients were too low for the sensitivity of the assay which was 39 ng/ml in-house). There was no obvious correlation with response both at baseline or post-treatment.

### DISCUSSION

The results of this randomized phase II study suggest a benefit by combining intravenous chemotherapy with intradermal injection of SRL172, heat-killed *Mycobacterium vaccae*, in patients with lung cancer. Combining cytotoxic drugs with immune-modulators is not a new concept (Mitchell, 1992) but there have been concerns that negative interactions might take place due to the myelo-suppressive properties of many cytotoxic drugs. Cytotoxic drugs also preferentially kill cells in division, a hallmark of an activated immune system, and therefore could inhibit immune responses. However, the particular combination of cytotoxic drugs used in this study is not very myelo-suppressive at the doses prescribed in lung cancer patients and therefore the mycobacterium was given with the chemotherapy.

The cytotoxic drugs were administered according to the standard protocol at 3 weekly intervals, whereas we decided empirically to follow a monthly schedule with the *Mycobacterium vaccae* because of previous vaccination protocols with SRL172 designed for patients with melanoma, prostate cancer, tuberculosis and AIDS. Thus, one intradermal SRL172 injection was given concommitantly with the chemotherapy, the second boost was given one week after chemotherapy and the third was given 2 weeks after the chemotherapeutic drugs. Theoretically, the timing of the SRL172 injection in relation to the administration of chemotherapy may be relevant to any cytotoxic effects. Chemotherapeutic agents usually induce myelosuppression with a decrease in white blood cell count after 2 to 7 days with a rebound between 1 to 2 weeks. Thus the first 3 injections of SRL172 corresponded to different points in the chemotherapy cycle. The first injection was just before chemotherapy covering the time when early apoptosis may have been induced by chemotherapy (24 hours) (Ellis et al, 1997). The second injection (day 7 post

*© 2000 Cancer Research Campaign* 

**Table 4** Serum cytokine levels at baseline and post-treatment

| Cytokine | Response to treatment | Baseline (mean) | Post-treatment (1 month) | *P* value* |
|---|---|---|---|---|
| | Resp Chemo | 4.5 (< 1–13.6) | 1.9 (< 1–5.9) | 0.9 |
| | Chemo + SRL172 | 4.1 (< 1–11.7) | 1.0 (< 1–2.3) | 0.3 |
| γ-INF (pg/ml) | | 4.2 | 1.32 | 0.15 |
| | N.Resp Chemo | 13.3 (< 1–30.3) | 3.5 (< 1–12.1) | 0.1 |
| | Chemo + SRL172 | 1.3 (< 1–5.3) | 0.7 (< 1–2.65) | 0.99 |
| | | 8.5 | 2.3 | 0.1 |
| | Resp Chemo | 39.1 (33.2–45.7) | 38.6 (36.8–39.5) | 0.99 |
| | Chemo + SRL172 | 37.2 (33.9–41.4) | 42.8 (38.8–51.2) | 0.18 |
| TNF-α (pg/ml) | | 37.9 | 41.2 | 0.3 |
| | N.Resp Chemo | 43.6 (31.9–50.2) | 45.4 (35.9–57.4) | 0.56 |
| | Chemo + SRL172 | 49.1 (39.5–60.6) | 43.9 (28–50.7) | 0.62 |
| | | 45.8 | 44.8 | 0.9 |

The mean values for all responders (Resp) and non responders, (N.Resp) irrespective of the treatment are also shown for both cytokines (third, sixth, ninth and last line). *Paired Wilcoxon signed rank test.

chemotherapy) may have corresponded to the time where antigen shedding from the tumour took place and the third injection (day 15 post chemotherapy) should have covered the period of bone marrow and lymphocyte recovery.

Other workers have suggested that SRL172 may be a non-specific immunomodulator and that it may promote a predominantly Th1 type (cell-mediated) response (Grange et al, 1995). For example, in a study of 10 patients with advanced prostate cancer who were treated with SRL172 alone, PSA levels were found to decrease in 2 out of 10 patients as a result of treatment (Hrouda et al, 1998). The response in these patients correlated with increased intracellular IL-2 production in lymphocytes, a finding reported by the same group to occur in advanced melanoma patients (Maraveyas et al, 1999). However, the mechanism of the possible anti-tumour effect is unclear. It has been reported that patients with malignant disease have defective Th1 cell function in association with elevated Th2 cell levels (Pellegrine et al, 1996); thus a change in the balance towards improved Th1 function may be of critical importance to the success of immunotherapy in cancer.

In the present study, we measured serum cytokines only. Serum cytokines levels of gamma-IFN and TNF-α were not different between the two treatment groups indicating that there was no bias of selection for these cytokine levels. However, there was a lower TNF-α serum level in the responders compared to the non-responders and at present we do not know if this could have prognostic significance. There was no correlation with response although serum cytokine changes could have been missed because of the timing of the assay or the lack of sensitivity of the assay as in the case of IL-10. We have just completed a subsequent study in mesothelioma patients treated with chemotherapy and SRL172. We found that the patients who responded to treatment had decreased level of IL-4 producing T lymphocytes and activation of natural killer (NK) cells (assessed by CD69 expression) with concomitant increase in NK cell number (BES, in preparation). However, we did not find an increase of IL-2-producing T cells in the responder patients. At this stage, we do not know if the immunological changes contributed to or were a consequence of tumour regression, or if they were simply due to SRL172 injection with no role in the anti-tumour process or, even, due to chemotherapy alone. Therefore the mode of action of SRL172 is still under investigation in our laboratory.

Reports of possible synergy between chemotherapy and immunotherapy date back to the early 1980s when the work of Hanna and Key demonstrated increased survival in guinea pigs bearing syngeneic L10 hepatocarcinoma cells when cytotoxic drugs were administered at the peak of the inflammatory response following tumour vaccination. They hypothesized that drug cytotoxicity was enhanced in the tumour if tumour architecture was disrupted by the inflammatory reaction, allowing better access of the drugs into the tumour (Hanna and Key, 1982). Another possibility is that SRL172 may possess some true vaccine properties, and may express cross-reactive antigens with cancer cells. Obvious candidates for such cross-reactive antigens are heat-shock proteins (hsp) which are highly conserved across species; thus mycobacterial hsp are likely to show homology with those known to be present on human tumour cells. Some hsps are highly expressed in cancer cells compared with normal cells (Ferrarini et al, 1992) and it might be anticipated that the hsp expression levels would increase after treatment with chemotherapy. This possibility is at present under investigation in our laboratory.

This study encouraged a large phase III randomized trial to test the hypothesis that there may indeed be a clinical interaction between SRL172 and combination chemotherapy in patients with non-small-cell lung cancer. Such a trial is now underway with a recruitment of 418 patients completed.

### ACKNOWLEDGEMENTS

We are grateful to SR Pharma plc for support.

### REFERENCES

Al-Moundhri M, O'Brien M and Souberbielle BE (1998) Immunotherapy and lung cancer. *Br J Cancer* **78**: 282–288

Ellis PA, Smith IE and Hardy JR (1995) Symptom relief with MVP (mitomycin C, vinblastine and cisplatin) chemotherapy in advanced non-small-cell lung cancer. *Br J Cancer* **71**: 366–370

Ellis PA, Smith IE, McCarthy K, Detre S, Salter J and Dowsett M (1997) Preoperative chemotherapy induces apoptosis in realy breast cancer. *Lancet* **349**: 849

Ferrarini M, Heltai S, Zocchi MR and Rugarli C (1992) Unusual expression of and localisation heat-shock proteins in human tumour cells. *Int J Cancer* **51**: 613–619

© 2000 Cancer Research Campaign

Grange JM, Stanford JL and Rook GAW (1995) Tuberculosis and cancer: parallels in host responses and therapeutic approaches? *Lancet* **345**: 1350–1352
Hanna MG and Key ME (1982) Immunotherapy of metastases enhances subsequent chemotherapy. *Science* **217**: 367–369
Hrouda D, Baban B, Dunsmuir WD, Kirby RS and Dalgleish AG (1998) Immunotherapy of advanced prostate cancer: a phase I/II trial using *Mycobacterium vaccae* (SRL172). *Br J Urol* **82**: 568–573
Maraveyas A, Baban B, Kennard D, Rook GAW, Westby M, Grange JM, Lydiard P, Stanford JL, Jones M, Selby P and Dalgleish AGD (1999). Possible improved survival of patients with stage IV AJCC melanoma receiving SRL172 immunotherapy: correlation with induction of increased levels of intracellular interleukin-2 in peripheral blood lymphocytes. *Ann Oncol* **10**: 817–824
Mitchell MS (1992) Chemotherapy in combination with biomodulation: a 5 year experience with cyclophosphamide and IL-2. *Semin Oncol* **19** (supp 2): 80–87
Pellegrine P, Berhelia AM and Del Beato T (1996) Dysregulation of Th1 and Th2 subsets of CD4+ cells in peripheral blood of colorectal patients and involvement in cancer establishment and progression. *Cancer Immunol Immunother* **42**: 1–8
Webb A and O’Brien MER (1998) Where do we go with new expensive treatments in NSCLC? *Br J Cancer* **78**(2): 159–162

© 2000 Cancer Research Campaign 
